# Inclisiran—Silencing the Cholesterol, Speaking up the Prognosis

**DOI:** 10.3390/jcm10112467

**Published:** 2021-06-02

**Authors:** Sylwester Rogula, Ewelina Błażejowska, Aleksandra Gąsecka, Łukasz Szarpak, Milosz J. Jaguszewski, Tomasz Mazurek, Krzysztof J. Filipiak

**Affiliations:** 1 1st Chair and Department of Cardiology, Medical University of Warsaw, Banacha 1a, 02-097 Warsaw, Poland; sylwesterrogula@o2.pl (S.R.); s073920@student.wum.edu.pl (E.B.); tmazurek@kardia.edu.pl (T.M.); krzysztof.filipiak@wum.edu.pl (K.J.F.); 2 Maria Sklodowska-Curie Białystok Oncology Centre, Ogrodowa 12, 15-027 Białystok, Poland; lukasz.szarpak@gmail.com; 3 Maria Sklodowska-Curie Medical Academy in Warsaw, Solidarności 12, 03-411 Warsaw, Poland; 4 1st Department of Cardiology, Medical University of Gdańsk, Dębinki 7, 80-211 Gdańsk, Poland; jamilosz@gmail.com

**Keywords:** atherosclerosis, cardiovascular disease, hypercholesterolemia, inclisiran, siRNA

## Abstract

The reduction of circulating low-density lipoprotein-cholesterol (LDL-C) is a primary target in cardiovascular risk reduction due to its well-established benefits in terms of decreased mortality. Despite the use of statin therapy, 10%–20% of high- and very-high-risk patients do not reach their LDL-C targets. There is an urgent need for improved strategies to manage dyslipidemia, especially among patients with homozygous familial hypercholesterolemia, but also in patients with established cardiovascular disease who fail to achieve LDL goals despite combined statin, ezetimibe, and PCSK9 inhibitor (PCSK9i) therapy. Inclisiran is a disruptive, first-in-class small interfering RNA (siRNA)-based therapeutic developed for the treatment of hypercholesterolemia that inhibits proprotein convertase subtilisin–kexin type 9 (PCSK9) synthesis, thereby upregulating the number of LDL receptors on the hepatocytes, thus lowering the plasma LDL-C concentration. Inclisiran decreases the LDL-C levels by over 50% with one dose every 6 months, making it a simple and well-tolerated treatment strategy. In this review, we summarize the general information regarding (i) the role of LDL-C in atherosclerotic cardiovascular disease, (ii) data regarding the role of PCSK9 in cholesterol metabolism, (iii) pleiotropic effects of PCSK9, and (iv) the effects of PCSK9 silencing. In addition, we focus on inclisiran, in terms of its (i) mechanism of action, (ii) biological efficacy and safety, (iii) results from the ORION trials, (iv) benefits of its combination with statins, and (v) its potential future role in atherosclerotic cardiovascular disease.

## 1. Introduction 

Cholesterol is one of the major components of cellular membranes, which plays an important role in hormone and bile acid synthesis [1]. An increased level of circulating low-density lipoprotein-cholesterol (LDL-C) is the main risk factor for cardiovascular disease. Long-lasting hypercholesterolemia is a key factor in the development and progression of atherosclerosis, including coronary, carotid, and peripheral vessel disease, and has direct negative effects on the myocardium itself [2]. Atherosclerosis and related complications, including acute coronary syndromes, are the major cause of death worldwide [3]. Accordingly, there is a linear relationship between the reduction of cholesterol levels and improvements in clinical outcomes in patients suffering from hypercholesterolemia [4].

The reduction of LDL-C is the primary target for the reduction of cardiovascular risk and the best marker of clinical efficacy under lipid-lowering therapies due to its well-established benefits in terms of decreased mortality [5]. Hydroxymethylglutaryl-coenzyme A reductase inhibitors (statins) are the most commonly prescribed drugs for hypercholesterolemia treatment due to their well-established lipid-lowering effects [6]. However, 10%–20% of high- and very-high-risk patients do not reach their LDL-C targets despite statin therapy, indicating the urgent need for improved strategies to manage hypercholesterolemia in these patients [7]. The residual risk may be managed with further LDL-C reductions. According to ESC/EAS guidelines for the management of dyslipidemias, if the target level of LDL-C is not achieved with the maximum tolerated dose of statins, a combination of statins with ezetimibe is recommended. In addition, for secondary prevention in very-high-risk patients and for very-high-risk familial hypercholesterolemia (FH) patients who do not reach their LDL-C targets on the maximum tolerated doses of statin and ezetimibe, a combination with a proprotein convertase subtilisin/kexin type inhibitor (PCSK9i) is recommended [8]. The monoclonal antibodies, PCSK9is evolocumab, alirocumab, and bococizumab have been shown to reduce major adverse cardiovascular events when used as an add-on therapy to statins [9]. Simultaneous therapy with a statin and PCSK9i can reduce LDL-C levels by an additional 64%, in comparison to statins only, which is a larger LDL-C decrease than has ever been achieved with monotherapy [10]. The clinical benefits of evolocumab were assessed in the FOURIER trial, where evolocumab lowered LDL-C levels by 59% and reduced the risk of cardiovascular death, myocardial infarction (MI), or stroke by 20%, compared with a placebo. The clinical advantages of alirocumab were evaluated in the ODYSSEY Outcomes trial, where alirocumab lowered the LDL-C levels by 57% and reduced the risk of cardiovascular death, MI, stroke, and hospitalization for unstable angina by 15%, compared with the placebo [11].

Statin therapy may be associated with many side effects, such as myalgia or rhabdomyolysis, which are reasons for the discontinuation of statins. In fact, statin intolerance currently represents an essential reason for initiating treatment with ezetimibe and a therapeutic option complementary to ezetimibe—bempedoic acid. In statin-intolerant patients, bempedoic acid added to ezetimibe reduced LDL-C levels (−36.2%), as compared with a placebo (−1.8%) [12]. Moreover, bempedoic acid was well-tolerated with a frequency of muscle-related adverse events comparable to the placebo group [13]. However, the reduction of LDL-C levels with a combination of ezetimibe and bempedoic acid was not as spectacular as in the case of PCSK9is (64%) [10]. Although PCSK9is enabled the achievement of the target LDL-C level in the vast majority of patients, some patients seemed to be resistant to therapy, mostly due to (i) poor adherence, (ii) improper PCSK9i administration, and (iii) dermatological factors impairing PCSK9i absorption [14,15]. Furthermore, LDL-receptor deficient homozygous familial hypercholesterolemia (HoFH) patients respond poorly to PCSK9i therapy [16].

To circumvent the disadvantages of statins and PCSK9i, a novel approach to lipid-lowering therapy with inclisiran has been developed. Inclisiran is a first-in-class small interfering RNA (siRNA)-based therapeutic developed for the treatment of hypercholesterolemia. Inclisiran is a chemically modified siRNA that inhibits proprotein convertase subtilisin–kexin type 9 (PCSK9) synthesis, thereby upregulating a number of LDL-receptors on the hepatocytes, thus lowering the plasma LDL-C concentration [17]. In contrast to statins, which require daily oral administration, and PCSK-9 inhibitors, where one subcutaneous injection every 2–4 weeks is required, inclisiran decreases the LDL-C levels by over 50% with one dose every 6 months [18].

This review consists of two parts. In the first part, we summarize the general information regarding (i) the role of LDL-C in atherosclerotic cardiovascular disease (ASCVD), (ii) data regarding the role of PCSK9 in cholesterol metabolism, (iii) pleiotropic effects of PCSK9, and (iv) the effects of PCSK9 silencing. In the second part, we focus on inclisiran, discussing (i) its mechanism of action, (ii) its biological efficacy and safety, (iii) results from the ORION trials, (iv) benefits of its combination with statins, and (v) its potential future role in ASCVD.

## 2. LDL-Cholesterol Role in Atherosclerotic Cardiovascular Disease

Atherosclerosis is a chronic, progressive pathological process that involves endothelial damage, lipid abnormalities, activation of the immune system, transformation of monocytes to macrophages, vascular smooth muscle cell (VSMC) proliferation, and thrombosis [19]. ASCVD is a result of a long-lasting atherosclerosis progression. ASCVD develops and progresses sub-clinically, before manifesting as an acute coronary syndrome, acute ischemic stroke, or peripheral artery disease, which poses a challenge for healthcare systems worldwide both due to its direct economic impact and due to its detrimental effect on patients’ quality of life [20,21].

Lipid deposition in the vascular wall is one of the most important factors in the pathogenesis of atherosclerosis [22]. It has been established that low-density lipoproteins (LDL), which mainly contain cholesterol, accumulate in the intima and stimulate the expression of adhesion molecules and chemoattractants on the surface of endothelial cells. This process results in the activation of the circulating monocytes and their adhesion to the endothelium. After migration into the intima, monocytes differentiate into macrophages. Subsequently, they internalize and accumulate LDLs, which results in their transformation into the most characteristic cells of atherosclerosis, foam cells [23]. LDL receptors (LDL-Rs) play a vital role in the cellular metabolism of cholesterol. They identify and internalize native LDL-C [24], which is the major carrier of cholesterol to body tissues. An elevated plasma LDL-C level is a well-recognized risk factor for CVD. The measurement of LDL-C is routinely used for CVD risk assessment and risk management [25].

**Figure 1:**
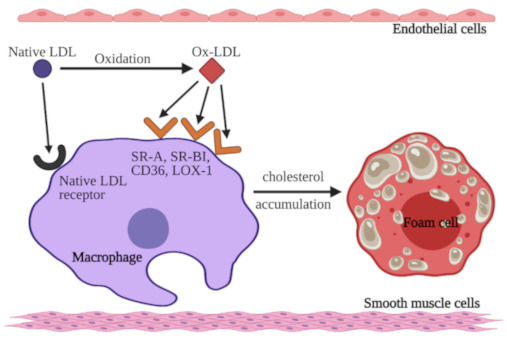
Mechanism of native low-density lipoprotein-cholesterol (LDL-C) and oxidized LDL (ox-LDL) uptake by macrophages. Native LDL-C binds to LDL receptors on macrophages or undergoes oxidation. Ox-LDL binds to specific receptors: scavenger receptor class A (SR-A), scavenger receptor class B type I (SR-BI), cluster differentiating 36 (CD36), and lectin-like oxidized LDL receptor-1 (LOX-1) on macrophages as well. Macrophages accumulate cholesterol from both native LDL and ox-LDL, which results in the formation of foam cells. Figure created with BioRender.com (accessed on 12 May 2021).

It has been proven that the incubation of LDLs with endothelium cells or smooth muscle cells could convert LDL into oxidized LDL (ox-LDL). Ox-LDL is the major modified form of native LDL because LDL particles are extremely sensitive to oxidative damage [26]. Native LDL is recognized and internalized by LDL-R. In contrast, ox-LDL is not able to bind with LDL-R and hence remains in the circulation. Scavenger receptors (SRs) are responsible for the recognition and internalization of ox-LDL [27]. As a result of these processes, cholesterol uptake by macrophages increases. SRs belong to a class of receptors expressed on various cells, such as endothelial cells, macrophages, and platelets. The SR family includes scavenger receptor class A (SR-A), cluster differentiating 36 (CD36), scavenger receptor class B type I (SR-BI), cluster differentiating 68 (CD68), scavenger receptor for phosphatidylserine and oxidized lipoprotein (SR-PSOX), and lectin-like oxidized LDL receptor-1 (LOX-1), among others [25]. In contrast to LDL-Rs, SRs are not downregulated by increased levels of intracellular cholesterol [28]. As mentioned before, macrophages use SRs to internalize ox-LDL. This process leads to remarkable intracellular cholesterol accumulation. As a result, macrophages are converted to foam cells and promote the development of fatty streak lesions, a marker of early atherosclerosis [29,30]. The mechanisms of LDL-C and ox-LDL uptake by macrophages in the vessel wall are presented in Figure 1.

Ox-LDL exerts proatherosclerotic properties by promoting (i) the activation and dysfunction of endothelial cells, (ii) the migration and proliferation of VSMCs, and (iii) platelet activation [31]. Ox-LDL promotes platelet activation, and ox-LDL-positive platelets further interact with the endothelium, thus promoting vascular inflammation and endothelial dysfunction [32,33]. Elevated ox-LDL levels may be more causally associated with cardiovascular events than LDL due to its central role in atherosclerotic plaque biology [34]. Considering this, ox-LDL levels may have the potential to improve risk prediction beyond LDL-C. Indeed, a recent meta-analysis revealed that elevated serum ox-LDL is associated with an increased risk of CVD events [35].

The adhesion of monocytes and leukocytes to the endothelium is dependent on both chemokines and adhesion molecules. In the endothelium, ox-LDL increases the expression of adhesion molecules (vascular and intercellular cell adhesion molecule-1, P-selectin, and E-selectin) [36,37]. In addition, ox-LDL is a chemoattractant molecule for monocytes [38]. Ox-LDL also has an impact on nitric oxide (NO) production due to (i) inhibition of the expression of endothelial NO synthase, and (ii) inactivation of NO by increasing reactive oxygen species production [39,40,41]. NO exerts antiatherogenic properties through endothelium-mediated vasodilation. An increased ox-LDL plasma level both antagonizes the NO production and stimulates the generation of endothelin-1, which increases the vascular tone [31]. Finally, ox-LDL activates nuclear factor kappa B (NF-κB), which further modulates the expression of proinflammatory genes and triggers the apoptosis of endothelial cells [25]. Ox-LDL is also involved in vascular remodeling by increasing the expression of metalloproteinases 1 and 3, which are responsible for intercellular matrix degradation [42].

LDL-C levels have long been described as a circulating biomarker reflecting general cardiovascular risk. Hence, it remains the most important therapeutic target in the primary and secondary prevention of CVD [43]. However, a high concentration of ox-LDL in the vascular wall may also be associated with an increased risk of future plaque rupture [44]. In high concentrations, ox-LDL (i) enhances LOX-1 expression, (ii) induces apoptosis of VSMCs, which may lead to plaque destabilization, and (iii) increases collagen synthesis by VSMCs and fibroblasts, leading to the formation of a fibrous capsule, thus converting the fatty streak to an atheroma [45,46,47]. Hence, the reduction of circulating ox-LDL seems to be the most important target in preventing ASCVD development and progression.

## 3. PCSK9’s Role in Cholesterol Metabolism

LDL-C particles are produced as a result of circulating lipoprotein maturation. LDL-C consists of a high amount of cholesterol, a low amount of triglycerides, and the apoB-100 protein, which acts as a ligand for binding to LDL-C receptors. The role of LDL-C receptors is very important in ASCVD development. The reduction of LDL receptor exposure on the hepatocyte cell membrane increases the levels of circulating LDL-C [48,49].

The LDL-C and LDL-receptor complex is internalized and undergoes processing by the endosomal–lysosomal system. At the lysosome, LDL particles are decomposed into cholesterol and triglycerides and subsequently transported into the cytosol [49]. Similarly, the LDL receptor can either be recycled back to the cell membrane or digested [10].

The lifespan of LDL receptors is modulated by PCSK9. Circulating PCSK9 in its active form binds to LDL receptors, promoting their internalization and degradation in lysosomes. PCSK9 disrupts the normal recycling of LDL receptors. A reduction in cell-surface receptor density leads to raised plasma levels of LDL-C [50,51]. Conversely, inhibition of PCSK9 leads to the upregulation of both recycling and the expression of LDL-Rs at the cell surface. As a consequence, clearance of LDL-C from the bloodstream increases, and the LDL-C plasma concentration substantially decreases [10].

## 4. Pleiotropic Effects of PCSK9

Preclinical studies have shown that PCSK9 exerts pleiotropic effects beyond plasma LDL regulation and seems to play a crucial role in atherogenesis. PCSK9 is expressed on various cells types that are involved in atherosclerosis: endothelial cells [52], VSMCs [53], and macrophages [54]. Overexpressed PCSK9 accumulates in the arterial wall and directly affects atherosclerosis lesion size and composition, independently of plasma lipid and lipoprotein concentrations [55]. In murine models of the overexpression of either normal or gain-of-function PCSK9, increased atherosclerotic plaque size was observed [56,57]. The Chinese Multi-provincial Cohort Study, which involved 643 participants free of cardiovascular disease at baseline showed that PCSK9 levels are associated with the progression of atherosclerosis, as reflected by the total plaque area, independently of plasma LDL-C concentrations [58]. PCSK9 protein has an impact on the metabolism of triglyceride-rich lipoprotein (TRL)—highly atherogenic particles associated with the initiation and propagation of atherosclerosis. This connection could provide the mechanism by which therapeutic antagonism of PCSK9 may reduce the risk of ASCVD [59].

The migration of monocytes is a key element in the development of atherosclerosis lesions, where monocyte-derived macrophages contribute to a local pro-inflammatory environment [60,61]. Treatment of familial hypercholesterolemia with PCSK9i markedly reduced the migration capacity of monocytes, which was associated with anti-inflammatory effects [62]. In the apolipoprotein E (apoE) knockout mouse model, it has been shown that atheromatous plaque was enriched with human PCSK9 and Ly6C(hi)—inflammatory monocytes. Human PCSK9 increased the infiltration of the inflammatory Ly6C(hi) monocytes into the plaque and their differentiation into macrophages, hence altering the plaque morphology [63]. Moreover, atherosclerotic apoE-knockout mice with silenced tissue PCSK9 expression presented the decreased expression of vascular inflammation regulators (i.e., tumor necrosis factor α (TNF-α), interleukin 1 (IL-1), Toll-like receptor 4 (TLR4), and NF-κB) and of monocyte chemoattractant protein-1 (MCP-1), as well as the decreased accumulation of macrophages in aortic plaques [64]. Likewise, PCSK9 inhibition in cultured VSMCs and in mice significantly decreased the expression of vascular cell adhesion molecule 1 (VCAM-1), thus reducing monocyte recruitment into the plaque [65].

As mentioned before, oxidation of LDL in the subendothelial space by locally produced reactive oxygen species may contribute to atherogenesis [66]. Transfection of EC and VSMC cultures with PCSK9 siRNA decreased the production of reactive oxygen species by 30% and 50%, respectively, and reduced the expression of NADPH oxidase—a major enzyme that generates reactive oxygen species—which suggests a direct anti-oxidative effect of PCSK9-inhibition [52].

**Figure 2:**
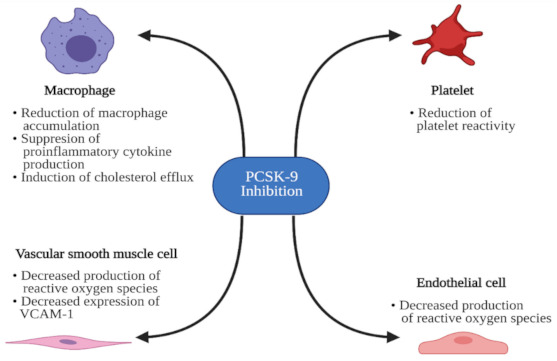
Effects of proprotein convertase subtilisin/kexin type 9 (PCSK9) inhibition on the crucial cells participating in atherosclerosis development. VCAM-1, vascular cell adhesion molecule 1. Figure created with BioRender.com (accessed on 12 May 2021).

Increased PCSK9 levels were also associated with carotid intima-media wall thickness (CIMT) independently of traditional cardiovascular risk factors, including gender, hypertension, smoking, LDL-C, triglycerides, Lp(a), obesity, and biomarkers of inflammation [67,68,69]. In asymptomatic patients with familial hypercholesterolemia, serum PCSK9 concentrations were independently predictive of coronary artery calcification (CAC). PCSK9 serum levels were significantly higher in subjects with the highest absolute CAC scores [70]. Moreover, PCSK9 concentrations were linearly associated with a higher necrotic core fraction in coronary plaque [71]. Effects of PCSK9 inhibition on the crucial cells participating in atherosclerosis development are presented in Figure 2. Altogether, the role of PCSK9 goes beyond the regulation of circulating lipid levels, and its inhibition may exert pleiotropic positive effects in patients with an increased risk of CVD.

## 5. Silencing PCSK9

The first sign of the future therapeutic role of PCSK9 inhibition was observed in two French families suffering from autosomal-dominant hypercholesterolemia [72]. Observational cohort and familial studies have shown that PCSK9 gain-of-function mutations are associated with an increased risk of ASCVD events [73,74,75]. Subsequently, a novel therapeutic approach to reducing ASCVD has emerged. In order to increase the number of hepatic LDL receptors, targeted inhibition of PCSK9, facilitating LDL reuptake from the bloodstream, has been introduced.

Evolocumab, alirocumab, and bococizumab are monoclonal antibodies that bind PCSK9 and inhibit its interaction with the LDL receptor. Recently, PCSK9is have emerged as an excellent therapeutic option for lowering LDL-C, either in monotherapy or in combination with statins [76]. Clinical studies have proven that PCSK9is provide a powerful reduction in circulating LDL-C levels. Remarkably, a meta-analysis of 71 randomized, placebo-controlled clinical trials proved that PCSK9is decrease the LDL-C serum levels by 50.7%, as compared to placebos [77]. PCSK9i also have multiple pleiotropic effects, including the stabilization of atherosclerotic plaque, antiplatelet effects, antineoplastic effects, and anti-bacterial effects [78]. A positive outcome of the use of PCSK9is resulted in their approval in both the US and Europe for FH patients who are intolerant to statins or at high risk of ASCVD, who require an especially potent LDL-C-lowering treatment [79].

PCSK9is need to be administered subcutaneously every 2–4 weeks [80]. Although the rate of injection-site reactions and allergic reactions are similar to those of placebos [80], the need for relatively frequent subcutaneous administration decreases the patients’ adherence to therapy, and hence the treatment benefits [14]. Hence, there is another possibility of PCSK9 inhibition. Monoclonal antibodies target and block plasma PCSK9, whereas small interfering RNAs (siRNAs) are 20–30 nucleotide RNA particles that prevent the intracellular translation of PCSK9 messenger RNAs (mRNAs) to protein [81]. SiRNAs selectively and catalytically silence the translation of their complementary target mRNAs. They act in a sequence-specific way through the formation of effector RNA-inducing silencing complexes (RISCs) [82,83].

## 6. Inclisiran: Mechanism of Action

Inclisiran consists of two strands: a guide strand and a passenger strand. Since siRNAs are unstable and not cell-permeable, five types of chemical modifications have been used to decrease their susceptibility to degradation and provide efficient delivery to target cells. Duplex RNA contains one 2′-deoxy-, eleven 2′-fluoro-, and thirty-two 2′-*O*-methyl-modified nucleotides. Three terminal nucleotides within unconjugated ends of the RNA strands contain thiophosphodiester linkages. The 3′ end of the passenger strand is conjugated to a triantennary *N*-acetylgalactosamine (GalNAc) [84]. GalNAc is a potent ligand of the asialoglycoprotein receptor (ASGPR) expressed on hepatocytes. Comparison of the uptake between siRNA-GalNAc conjugated and unconjugated siRNA has been assessed in freshly isolated primary mouse hepatocytes. It has been shown that conjugation of GalNAc to the 3′ end of the passenger strand resulted in robust uptake, in contrast to the unconjugated siRNA, which showed little or no uptake by hepatocytes [85].

**Figure 3:**
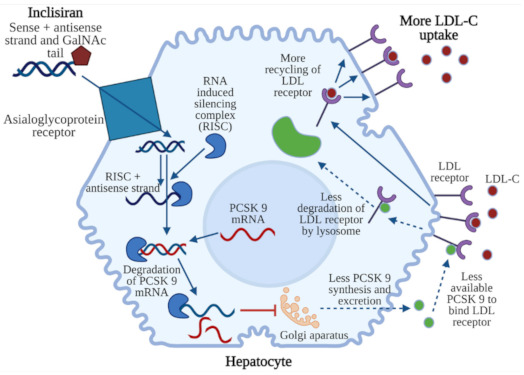
Mechanism of action of inclisiran. Inclisiran (sense + antisense strand connected with N-acetylgalactosamine) binds to a hepatocyte by means of an asialoglycoprotein receptor. The antisense strand is sliced by an RNA-induced silencing complex (RISC). The sense strand connects with mRNA of PCSK9, which leads to degradation of this complex. As a result, PCSK 9 synthesis in the Golgi apparatus and excretion are decreased. Less PCSK9 is available to bind LDL receptors, which leads to decreased degradation of LDL receptors in lysosomes. LDL receptors are not destroyed, which enables their re-exposure in the cell membrane and the uptake of more LDL cholesterol (LDL-C). Figure created with BioRender.com (accessed on 12 May 2021).

Inclisiran’s mechanism of action is based on RNA interference—the biological process in which double-stranded RNA silences the specific gene by triggering the complementary mRNA degradation [86]. When inclisiran enters the hepatocyte, through the interaction between GalNAc and ASGPR, the guide strand binds to the RNA-induced silencing complex (RISC). The combination of the guide strand and RISC binds PCSK9 mRNA and destroys it, preventing PCSK9 protein production. The mechanism of action of inclisiran is presented in Figure 3.

## 7. Biological Efficacy, Pharmacodynamic Properties, and Safety of Inclisiran

The first clinical study to show the safety and biological efficacy of inclisiran was a phase 1 trial—a randomized, single-blind, placebo-controlled study. A single dose of ALN-PCS (an inclisiran precursor molecule) or placebo was administered subcutaneously to healthy volunteers who were not receiving lipid-lowering treatment 30 days before screening and who had LDL-C levels higher than 3 millimoles per liter (mM/L). Inclisiran administration resulted in a rapid and dose-dependent reduction in plasma PCSK9 protein and LDL-C levels, with a dose-dependent duration of this effect. The maximum dose of inclisiran, 0.4 mg/kg, resulted in a mean 70% reduction in circulating PCSK9 plasma protein from baseline, compared to the placebo, with an individual maximum reduction of 84%. Administration of the highest dose resulted in a mean 40% reduction in LDL cholesterol levels from baseline, compared to the placebo, with an individual maximum reduction of 57%.

The ALN-PCS was safe and well-tolerated. Drug-related adverse events were moderate or mild. The rate of adverse events, including rash, headache, hiccups, cold symptoms, and infusion-site hematoma, was similar in the ALN-PCS and placebo groups (79% vs. 88%). Transient, mild, macular rash occurred on the first day after treatment with equal frequency and identical appearance in placebo and ALN-PCS-treated participants. This rash was probably a result of a premedication given to volunteers (oral corticosteroids, histamine receptor (H1 and H2) blockers, and paracetamol), which can cause skin flushing or skin rash. No clinically significant changes in any laboratory indices (liver function tests, creatine phosphokinase, C-reactive protein, and hematological measures) were noted. No significant alterations in the concentrations of the cytokines (for example, interferons α and γ (INF α and γ), IL 6, IL 12, TNF α, and granulocyte colony-stimulating factor (G-CSF)) were observed [87].

Patients with hypercholesterolemia and cardiovascular disease may suffer from other diseases, such as chronic kidney disease or diabetes. Data from preclinical studies in rats and cynomolgus macaques proved that subcutaneous injections of GalNAc-siRNAs demonstrated no impact on renal function [88]. The pharmacodynamic effects and safety profile of inclisiran were similar in study participants with normal and impaired renal function who were administered a single dose of inclisiran subcutaneously. Hence, dose adjustments of inclisiran are not required in these patients [89]. Furthermore, the presence of diabetes at baseline did not change the effectiveness of inclisiran in the management of dyslipidemias; LDL-C dropped between 28% and 52% in patients without diabetes, and between 28% and 55% in diabetics [90].

## 8. ORION—The Clinical Development Program

To further evaluate the safety and the efficacy of inclisiran, the clinical development program ORION was launched. ORION includes several completed and ongoing trials.

The ORION-1 trial (phase 2, multicenter, double-blind, placebo-controlled, multiple ascending-dose trial) evaluated the efficacy, safety, and tolerability of inclisiran in patients with elevated LDL-C levels who received maximum doses of lipid-lowering therapies (statins and ezetimibe). Different doses and dose regimens were evaluated during the trial. Patients with elevated LDL-C levels (1.8 mM for patients with and 2.6 mM for patients without a history of ASCVD, respectively) received one (at day 1) or two (at day 1 and day 90) doses of inclisiran, administered as a subcutaneous injection or placebo. The greatest reduction (52.6%) of LDL-cholesterol levels was achieved among patients who received two doses of 300 mg of inclisiran. A second 3000-mg dose administered at day 90 offered an additional 10% reduction in LDL-C levels. With both single-dose and two-dose regimens, the levels of PCSK9 were significantly reduced. At day 240, both PCSK9 and LDL cholesterol levels remained significantly lower in all inclisiran regimens, compared to baseline.

Adverse events were noted both among the inclisiran and placebo groups with identical frequency (76%), and most of them were mild or moderate. Severe adverse events (herpes zoster infection, influenza, or nasopharyngitis) occurred among 11% of patients who received inclisiran and 8% of patients who received the placebo. In three patients receiving inclisiran, hepatic enzyme levels were increased transiently [91].

One-year follow-up of the ORION-1 trial proved that inclisiran provided a long-term and durable decrease in LDL-C and PCSK9 levels. The greatest reduction in LDL-C (29.9% to 46.4%) and PCSK9 levels (43.1% to 60.5%) was obtained with two doses (at days 1 and 90) of inclisiran. Data from this trial suggest that the 300 mg dosing seemed to have the best efficacy, with the potential for dosing schedule of a dose every 6 months [92].

The ORION-10 and the ORION-11 (two randomized, double-blind, placebo-controlled, parallel-group, phase 3 trials) assessed the efficacy, safety, and adverse-event profiles of inclisiran for 18 months in patients with elevated LDL-C levels at high risk of CVD. Both trials revealed that the administration of inclisiran subcutaneously every 6 months lowered the LDL-C levels by approximately 50%. Furthermore, at 18 months, inclisiran reduced PCSK9 levels by 69.8% in the ORION-10 trial and by 63.6% in the ORION-11 trial. In addition, inclisiran decreased the levels of total cholesterol (TC), apolipoprotein B (ApoB), non-high-density lipoprotein cholesterol (non-HDL-C), triglycerides (TG), and lipoprotein (a) (Lp(a)), and increased HDL-C, compared with placebo. The adverse events described in the ORION-10 and the ORION-11 trials were mild and were observed with a similar rate in the inclisiran and placebo group. Only injection-site adverse events were more frequent in the inclisiran group than the placebo group (2.6% vs. 0.9% in the ORION-10 trial and 4.7% vs. 0.5% in the ORION-11 trial) [93].

The benefits of inclisiran therapy were noted especially among patients with heterozygous familial hypercholesterolemia (HeFH)—a genetic disorder characterized by elevated LDL cholesterol levels [94]. Subsequently, patients with HeFH who struggled with high LDL cholesterol levels (at least 2.6 mM) despite the treatment with a maximally accepted dose of statins were included in the ORION-9 trial. At 18 months, a reduction from baseline in the mean LDL cholesterol level of 50% or more was reported in 38% of patients in the inclisiran group and in 0.8% in the placebo group. In the inclisiran group, an LDL-C level reduction of 39.7% was observed, whereas in the placebo group an increase of 8.2% was noted. Adverse events were reported by 76.8% of participants in the inclisiran group and by 71.7% of participants in the placebo group and were moderate or mild. More patients in the inclisiran group had a protocol-defined injection-site reaction (17.0% vs. 1.7%), compared to the placebo group. Severe adverse events (nasopharyngitis, influenza, upper respiratory tract infection, back pain) occurred more often in the placebo group than the inclisiran group (13.8% vs. 7.5%). [95].

Inclisiran potentially represents a novel option to lower LDL-C levels in patients with homozygous familial hypercholesterolemia (HoFH). In these patients, long-term lipoprotein apheresis has hitherto been the only treatment to improve outcomes [96]. Moreover, inclisiran poses a promising medicine in HoFH patients with LDL receptor mutations in which PCSK9 inhibitors fail. Four patients suffering from HoFH with genetically confirmed mutations in both LDL receptor alleles were included in the ORION-2 pilot study. The ORION-2 was the open-label, single-arm, multicenter proof-of-concept study, which aimed to evaluate the efficacy and safety of inclisiran in patients with HoFH and confirm the dose and regimen for a subsequent phase 3 trial. All four participants achieved durable PCSK9 reductions on a background of high-intensity statins and ezetimibe (48.7% to 83.6% at day 90, 40.2% to 80.5% at day 180). The LDL level was durably lowered in three patients (11.7% to 33.1% at day 90, 17.5% to 37.0% at day 180). Inclisiran (300 mg) lowered PCSK9 and LDL-C in patients with HoFH without requiring dose or dosing regimen adjustments [97]. Base on these auspicious results, a larger phase 3 trial (the ORION-5 trial) is ongoing.

Inclisiran could also be a promising medication in patients at risk of stroke and myocardial infarction. To provide evidence regarding both the long-term safety and efficacy regarding hard clinical ASCVD endpoints of treatment with inclisiran, the ORION-4 trial was designed. ORION-4 is a double-blind randomized trial, which will assess the question of whether inclisiran reduces the risk of myocardial infarction and stroke in patients who have already had a major cardiovascular or cerebrovascular event, or who received interventional treatment due to ASCVD. The ORION-4 trial will enroll 15,000 participants aged ≥55 years, who will be randomized in a 1:1 ratio to receive inclisiran or placebo injections. Participants will be followed-up for five years [98].

Altogether, inclisiran appears to be a disruptive, efficient, and well-tolerated treatment, even in patients with comorbidities.

## 9. Inclisiran with Statins—A Promising Combination

Despite the efficacy of statins regarding the decreased LDL-C concentration and long-term CVD risk, a large number of patients do not achieve their therapeutic goals. Adherence to statin therapy remains a great challenge—evidence suggests that between 40%–75% of patients discontinue their statin therapy within one year after initiation [99]. Moreover, even after combining statins, ezetimibe, and PCSK9is, some patient subgroups (especially HoFH patients with LDL receptor mutation) still fail to achieve LDL-C targets. Twice-yearly injections of inclisiran could improve not only the efficacy of therapy but also patient adherence. The infrequent administration regimen is expected to increase the number of patients who maintain their therapeutic goals, especially in patients struggling to comply with daily (statins, ezetimibe) or biweekly pharmacotherapy (PCSK9i). Moreover, while PCSK9i acts at plasma level, inclisiran has an impact on the intracellular level (hepatocytes), without the involvement of the PCSK9 protein in the degradation of LDL receptors in lysosomes, which facilities liver regeneration and decreases the risk of hepatic damage [100]. The cost-effectiveness of inclisiran compared to PCSK9i remains an important issue. A cost-effectiveness analysis, adjusted for Australian healthcare, displayed that the price of inclisiran would have to be 60% lower than that of evolocumab [101]. According to Institute for Clinical and Economic Review (ICER), inclisiran should cost between USD 3600 and USD 6000 a year to be cost-effective, instead of the current price, which is ~USD 3898.75 for one syringe of 284 mg/1.5 mL [102,103].

**Figure 4:**
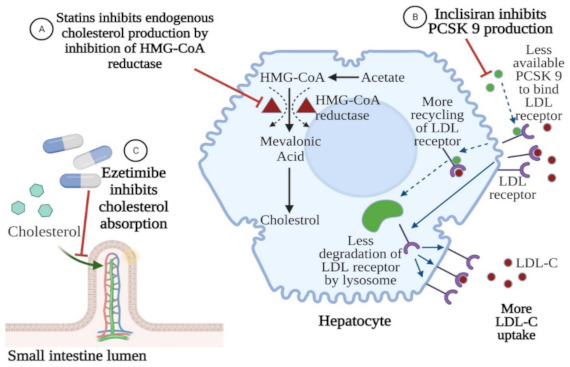
Compatible mechanisms of action of statins, ezetimibe, and inclisiran. Inclisiran inhibits PCSK9—proprotein convertase subtilisin-kexine type 9. LDL-C, low density lipoprotein cholesterol. Created with BioRender.com (accessed on 12 May 2021).

There is evidence suggesting that statins increase the circulating plasma PCSK9 levels in a dose-dependent manner. This phenomenon could decrease LDL cholesterol clearance from the bloodstream and hinder the effectiveness of statins as the dose is increased [104,105]. Inclisiran, which switches off the PCSK9 protein synthesis, may prevent the decline in LDL-C clearance. On the other hand, statins reduce triglyceride [106] and increase HDL cholesterol levels [107], whereas a 300-mg dose of inclisiran showed no significant difference with regard to HDL-C and triglyceride levels [108]. Moreover, statins display significant anti-inflammatory effects [109] and lower high sensitivity C-reactive protein (hsCRP) [110], which has been associated with decreased cardiovascular risk. PCSK9 inhibitors do not have a significant impact on hsCRP [111], although they decrease the level of circulating pro-inflammatory cytokines [54,64]. In addition, it has been shown that the combination of inclisiran with statins prevented 235 non-fatal myocardial infarctions and 114 coronary revascularization cases, compared with the use of a statin alone [101]. Hence, statins could complement inclisiran therapy when it comes to HDL-C concentration and hsCRP levels. The addition of PCSK9i:evolocumab or alirocumab to background statin therapy has also achieved a significant and sustained reduction in LDL-C levels and demonstrated an additional 48%–53% reduction of CV events [112]. Hence, the combination of either inclisiran or PCSK9 inhibitors with statins seems to be a promising strategy to combat hyperlipidemia. The compatible mechanisms of action of statins, ezetimibe, and inclisiran are shown in Figure 4.

Recently, newer agents from the class of angiopoietin-like 3 protein inhibitors (evinacumab) were introduced to reduce plasma total cholesterol, triglycerides, and low-density lipoprotein concentrations [113]. Evinacumab could also be combined with statins, as an alternative to PCKS9i, but the costs of treatment are higher than with PCKS9 modulators, the evidence-based experiences are lower, and this combination is preferentially dedicated to the subset of hypertriglyceridemia patients with familial hypercholesterolemia [114].

## 10. Potential Future Role of Inclisiran in Hyperlipidemia

At present, inclisiran is indicated in adults with primary dyslipidemia (heterozygous familial and non-familial) or mixed dyslipidemia in combination with other lipid-lowering therapies (e.g., statins) in patients unable to reach LDL-C goals with the maximum tolerated dose, or alone or in combination with other lipid-lowering therapies (statins/statins with ezetimibe/combined therapy of statins, ezetimibe, and PCSK9i) in patients who do not tolerate or have contraindications to statins. However, a number of ongoing trials will likely extend the indications of inclisiran to patients at high cardiovascular risk.

The safety and effectiveness of inclisiran in adult patients with HoFH will be evaluated in the ORION-5 (phase 3), a two-part, double-blind placebo-controlled/open-label, multicenter study (NCT03851705). The efficacy and safety of inclisiran in adolescents with HoFH will be evaluated in the ORION-13 (phase 3), a two-part, 1-year, double-blind inclisiran-versus-placebo, multicenter study (NCT04659863). The ORION-8 trial will assess the effect of long-term dosing of inclisiran in subjects at high cardiovascular risk—with ASCVD, ASCVD-risk equivalents (e.g., diabetes), HeFH or HoFH, and elevated LDL-C—who have completed any of the inclisiran phase 3 lipid-lowering studies (NCT03814187). The ORION-4 study aims to find out if inclisiran safely lowers the risk of myocardial infarction and strokes in patients who have already had a major cardiovascular or cerebrovascular event, or who were interventionally treated due to ASCVD (NCT03705234). Finally, ORION-3 is an extension trial of inclisiran compared to evolocumab in participants with cardiovascular disease and high LCL-C levels (NCT03060577).

Inclisiran is not only an effective PCSK9 modulator, but also an innovative medicine in the class of interfering-RNA therapeutics. Introduced in the treatment of hypercholesterolemia, it comes with other “sirans”, registered as the breakthrough designations patisiran (for a hereditary form of amyloidosis), givosiran (for acute hepatic porphyrias), and lumasiran (for primary hyperoxaluria type 1), along with even more “sirans” under investigation, such as fitusiran, cemdisiran, and ALN-TTRsc02.

The previously-mentioned angiopoietin-like protein-3 (ANGPTL3) inhibitors (evinacumab) recently introduced in familial hypercholesterolemia follow the same development route as PCSK9 modulators [115]. Evinacumab—the monoclonal antibody (like alirocumab/evolocumab)—was introduced as the first of its kind, but newer drugs based on RNA therapeutics technology, e.g., antisense oligonucleotides targeting mRNAs for ANGPTL3, are under investigation and some of them might be administered twice a year (like inclisiran). The study of vupanorsen, one of those (clinical trial TRANSLATE-TIMI 70) is in the recruitment phase, ending in 2022 (NCT04516291).

## 11. Conclusions

Silencing PCSK9 with inclisiran is a simple, effective, and well-tolerated approach to substantially improving outcomes in patients at high risk of cardiovascular and cerebrovascular events. The ongoing trials will probably extend the indications for inclisiran and clarify whether the infrequent administration of inclisiran (twice a year) in monotherapy and/or in combination with statins might further improve the prognosis and outcomes compared with other available lipid-lowering therapies, including PCSK9i.

## Data Availability

Not applicable.
